# [Corrigendum] UBE2T silencing suppresses proliferation and induces cell cycle arrest and apoptosis in bladder cancer cells

**DOI:** 10.3892/ol.2025.15326

**Published:** 2025-10-09

**Authors:** Yan Qing Gong, Ding Peng, Xiang Hui Ning, Xin Yu Yang, Xue Song Li, Li Qun Zhou, Ying Lu Guo

Oncol Lett 12: 4485–4492, 2016; DOI: 10.3892/ol.2016.5237

Following the publication of the above paper, it was drawn to the Editor's attention by a concerned reader that, for the cell proliferation assay experiments shown in Fig. 3A, the Control data panels shown for days 1–5 were apparently matching with the Control data panels shown for days 1–5 in Fig. 2B in a paper submitted to the journal *Oncotarget* at around the same time, even though the experiments were reported to have been performed using different cell lines (the 5637 and T24 cell lines, respectively).

After having re-examined this figure, the authors have realized that they inadvertently incorporated the incorrect set of data into Fig. 3A in the above paper. The revised version of Fig. 3, now showing the correct data for the Control data panels for days 1–5 in Fig. 3A, is shown on the next page; an amended version of Table I is also presented on the next page, now featuring the correct numerical data for the cell counts for the control data, and the fold changes in the cell counts. The authors wish to emphasize that the errors made in terms of the assembly of the data in this Figure and this Table did not affect the overall conclusions reported in the paper. The authors are grateful to the Editor of *Oncology Letters* for granting them this opportunity to publish a Corrigendum, and apologize to both the Editor and the readership for any inconvenience caused.

## Figures and Tables

**Figure 3.**
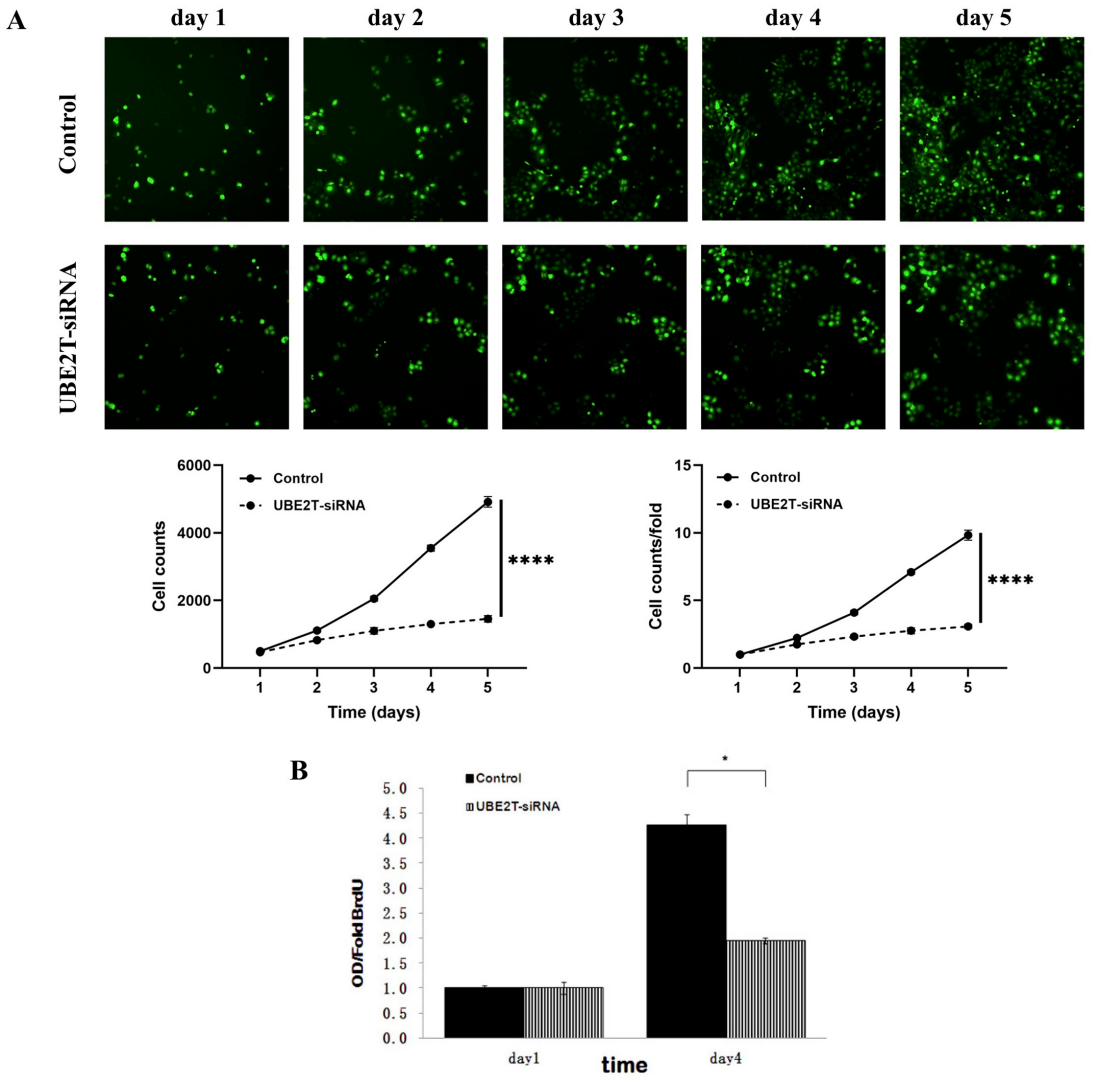
Lentivirus-mediated knockdown of UBE2T inhibited the proliferation of 5637 cells. (A) UBE2T knockdown attenuated 5637 cell growth potential *in vitro*. Cell growth was measured via multiparametric high-content screening every day for five days. Data are presented as the mean ± standard deviation. (B) The DNA synthesis rate was slowed in response to UBE2T downregulation, as analyzed by BrdU incorporation assay on the 1st and 4th days. (Control vs. UBE2T-siRNA, *P<0.05). UBE2T, ubiquitin-conjugating enzyme E2T; siRNA, small interfering RNA.

**Table I.** Cell numbers and growth rate as counted by cellomics.

	Cell count		Fold change in cell count	
		
Time	Control	UBE2T-siRNA	Control	UBE2T-siRNA
Day 1	500±2.65	473.0±36.29	1.00±0.00	1.00±0.00
Day 2	1,114±45.08	826.3±39.70	2.23±0.10	1.75±0.06
Day 3	2,055±71	1,103.3±97.11	4.11±0.16	2.33±0.14
Day 4	3,546±89.67	1,302.3±14.50	7.09±0.15	2.76±0.22
Day 5	4,913±157.81	1,457.3±93.22	9.83±0.36	3.09±0.15

Fold change in cell count was defined as cell count of Nth day / cell count of 1st day, where N=2, 3, 4 or 5.

